# Scientific Items

**Published:** 1878-08-20

**Authors:** 


### *Scientific Items.*

### FORZEN AIR.

M. Carelletet inclosed in a strong glass tube, dry air free from carbolic acid; he cooled with protoxide of nitrogen the upper part of the tube only. When the pressure was 200 atmospheres, streams of liquid air were seen flowing down the lower parts. When they met the mercury they seemed to turn back.

At 310 atmospheres, the mercury being in contact with the cooled part of the tube, was frozen, and on quickly removing the refrigerating apparatus, it was seen to be covered with what was probably *frozen air*.

### DYNAMITE.

Mr. Starr, in the minutes of the Connecticut State Board of Agriculture, says:

"I know but little about it (dynamite) except from results shown on my fields. A Mr. Parmelee, who makes it his business to blow up rocks with dynamite, passing my place, I asked him to experiment in one of my fields, which I proposed to clear of rocks. There was a large number of such as could not be blasted with powder, and I asked him what he could do. I said, 'I want you to experiment, and if you satisfy me I will let you work for a day or two.' I pointed to a rock 10½ feet long, 5½ wide, and 9 or 10 inches thick; such a rock as would be difficult to blast with powder. I took out my watch, and in 7½ minutes the rock was in atoms. I directed him to another larger rock, a shallow one, and he destroyed it in about the same time. I then set him at work during the afternoon, and I was so well pleased with the result that day, that I allowed him to work 2½ days on my farm, for which I paid him $80. I took care to have as few of my men with him as possible, for fear of accident. I am free to say that the same number of men, judging from the experience I have had in blasting for six years, could not have done in a month what he did in 2½ days."

### PREVALENCE OF OUR COMMON DISEASES IN JAPAN.

Dr. Stuart Eldridge, of Yokohama, a distinguished physician, who has had a long and varied experience in this country in hospital work and as an active practitioner, has kindly furnished me with the following data at my request: "Scarlet fever almost unknown, never epidemic. Diphtheria almost unknown, never epidemic. Severer forms of bowel disease, such as dysentery and chronic diarrhœa, very rare. Malarial diseases of a severe nature, uncommon; even the milder forms in most localities not common. Typhoid and typhus rarely epidemic, the latter uncommon."

With these facts before us, let us examine the conditions of living among these people. It is well known that their houses are so arranged that the winds blow through them from one end to the other. In summer they are entirely open. Prof. E. S. Morse,

*in Popular Science Monthly for Jan.*

### METALS IN THE SUN.

Out of the fifty-one metals with which we are acquainted here, more than 30 have, by means of the spectroscope, been discovered in the sun, and a distinguished scientist expresses the opinion that the sun consists wholly of metallic substances.

It is said that in China the telephone is used on a large scale. Very convenient, as the nature of the Chinese language prevents the use of the telegraph.

